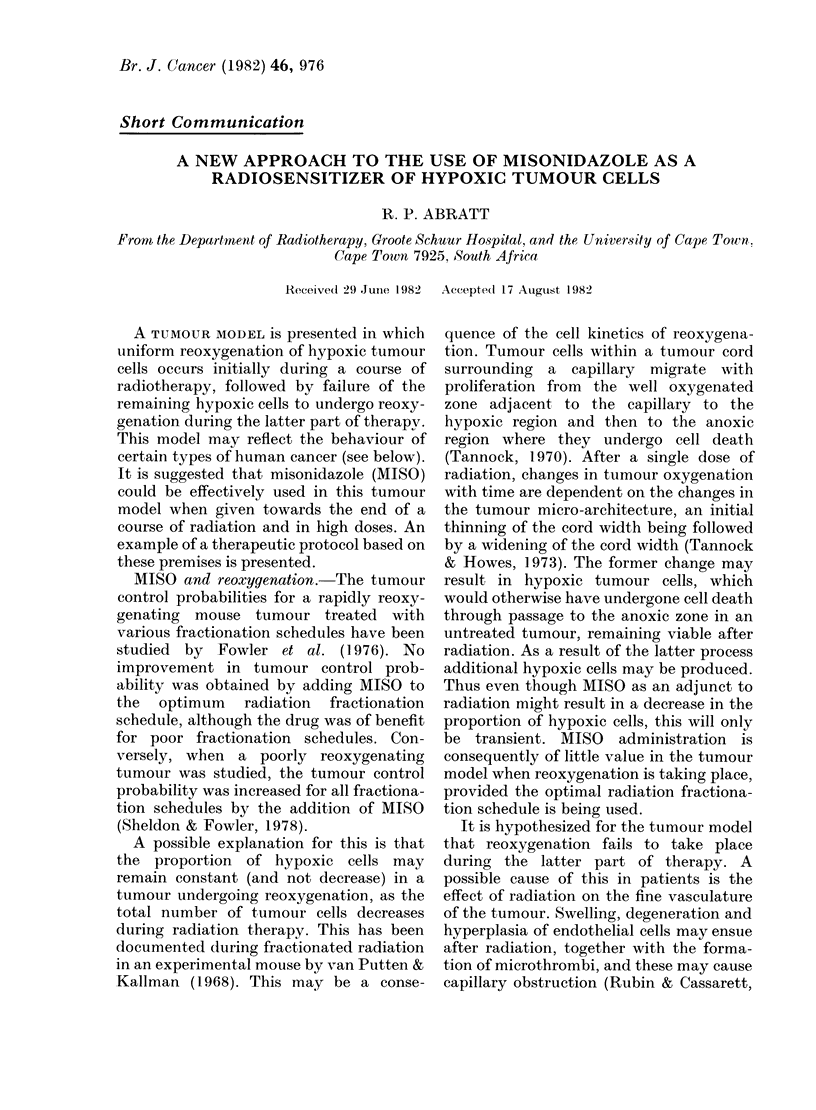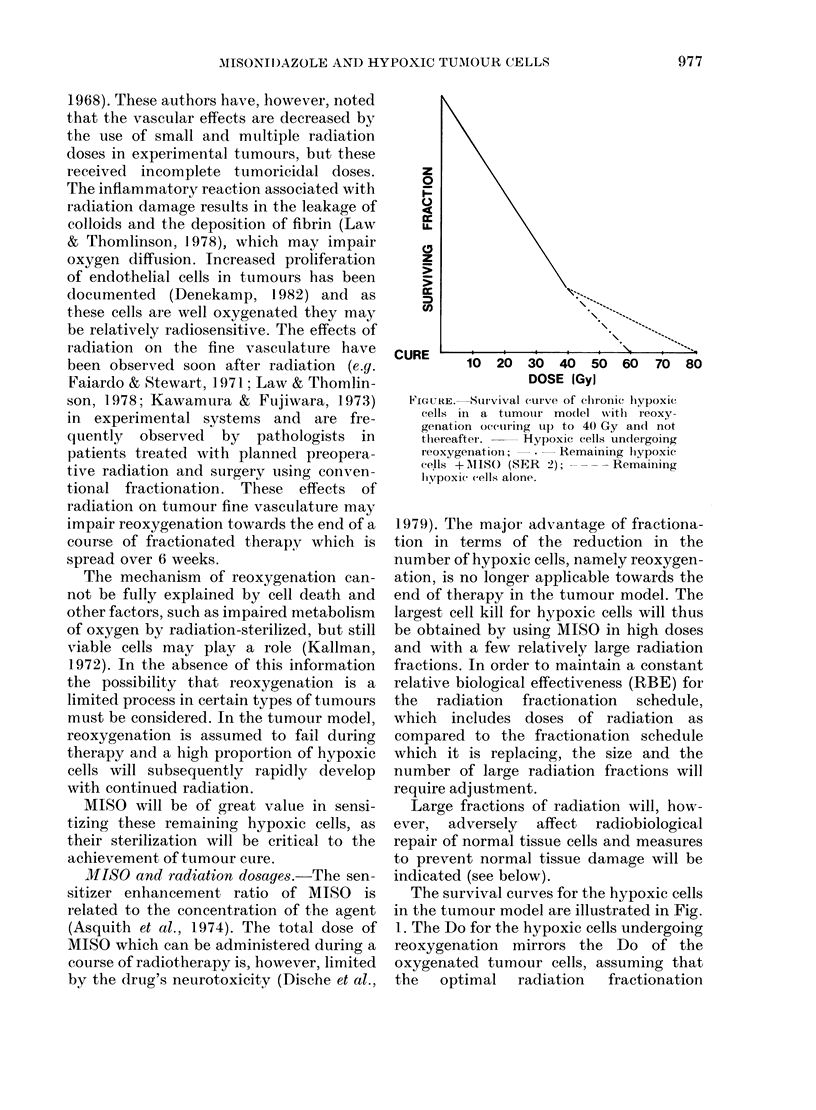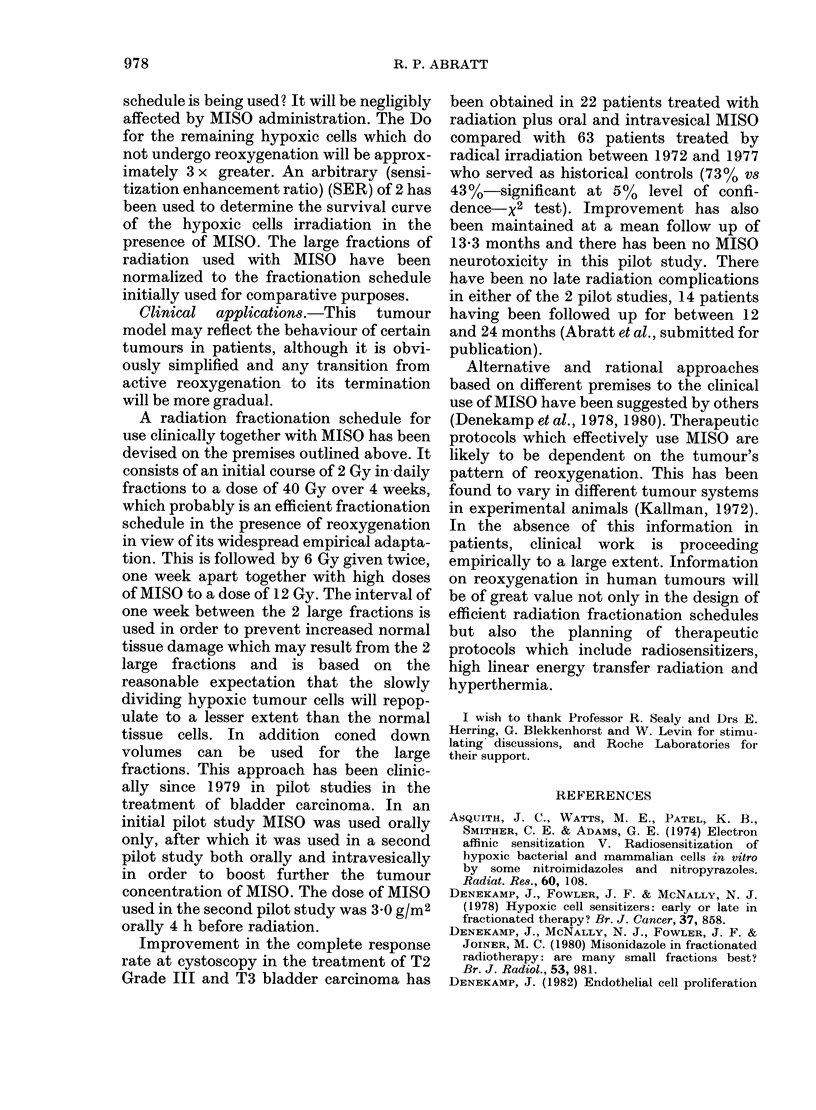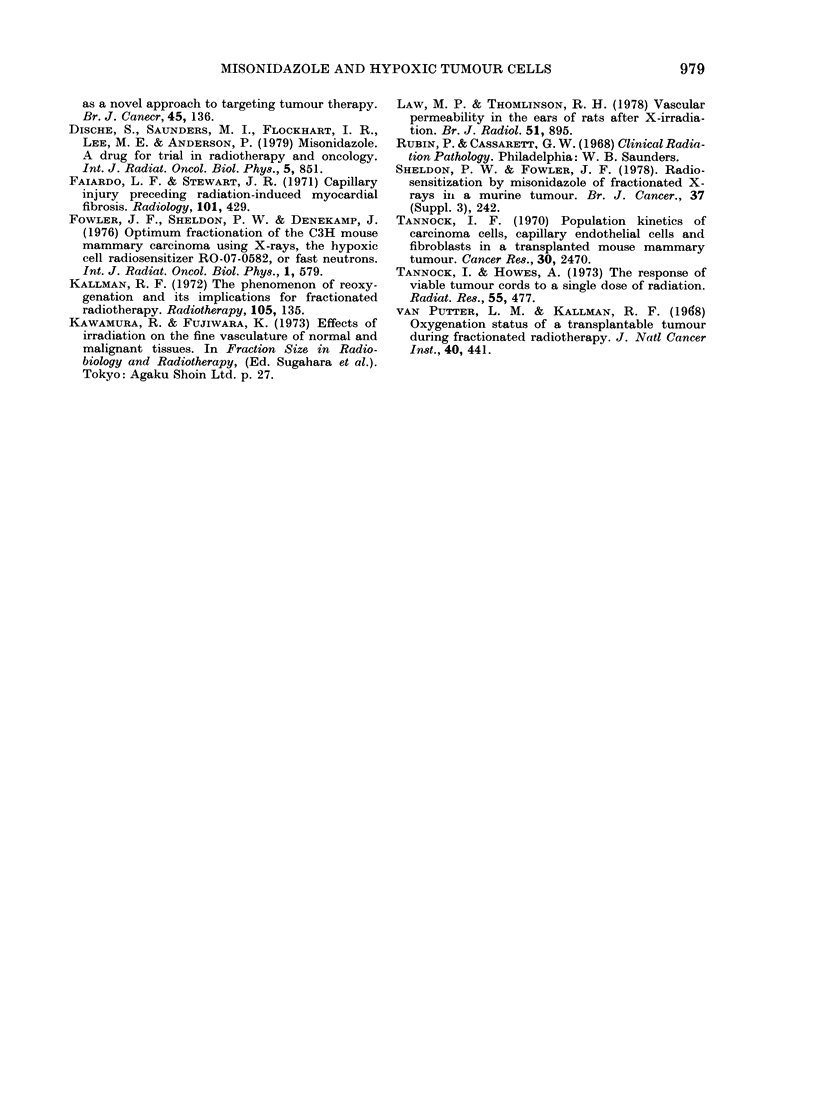# A new approach to the use of misonidazole as a radiosensitizer of hypoxic tumour cells.

**DOI:** 10.1038/bjc.1982.311

**Published:** 1982-12

**Authors:** R. P. Abratt


Br. J. Cancer (1982) 46, 976

*Short Communication*

### A NEW APPROACH TO THE USE OF MISONIDAZOLE AS A RADIOSENSITIZER OF HYPOXIC TUMOUR CELLS

R. P. ABRATT

*From the Department of Radiotherapy, Groote Schuur Hospital, and the University of Cape Town, Cape Town 7925, South Africa*

Received 29 June 1982 Accepted 17 August 1982

A TUMOUR MODEL is presented in which uniform reoxygenation of hypoxic tumour cells occurs initially during a course of radiotherapy, followed by failure of the remaining hypoxic cells to undergo reoxygenation during the latter part of therapy. This model may reflect the behaviour of certain types of human cancer (see below). It is suggested that misonidazole (MISO) could be effectively used in this tumour model when given towards the end of a course of radiation and in high doses. An example of a therapeutic protocol based on these premises is presented.

MISO *and reoxygenation.*—The tumour control probabilities for a rapidly reoxygenating mouse tumour treated with various fractionation schedules have been studied by Fowler *et al.* (1976). No improvement in tumour control probability was obtained by adding MISO to the optimum radiation fractionation schedule, although the drug was of benefit for poor fractionation schedules. Conversely, when a poorly reoxygenating tumour was studied, the tumour control probability was increased for all fractionation schedules by the addition of MISO (Sheldon & Fowler, 1978).

A possible explanation for this is that the proportion of hypoxic cells may remain constant (and not decrease) in a tumour undergoing reoxygenation, as the total number of tumour cells decreases during radiation therapy. This has been documented during fractionated radiation in an experimental mouse by van Putten & Kallman (1968). This may be a consequence of the cell kinetics of reoxygenation. Tumour cells within a tumour cord surrounding a capillary migrate with proliferation from the well oxygenated zone adjacent to the capillary to the hypoxic region and then to the anoxic region where they undergo cell death (Tannock, 1970). After a single dose of radiation, changes in tumour oxygenation with time are dependent on the changes in the tumour micro-architecture, an initial thinning of the cord width being followed by a widening of the cord width (Tannock & Howes, 1973). The former change may result in hypoxic tumour cells, which would otherwise have undergone cell death through passage to the anoxic zone in an untreated tumour, remaining viable after radiation. As a result of the latter process additional hypoxic cells may be produced. Thus even though MISO as an adjunct to radiation might result in a decrease in the proportion of hypoxic cells, this will only be transient. MISO administration is consequently of little value in the tumour model when reoxygenation is taking place, provided the optimal radiation fractionation schedule is being used.

It is hypothesized for the tumour model that reoxygenation fails to take place during the latter part of therapy. A possible cause of this in patients is the effect of radiation on the fine vasculature of the tumour. Swelling, degeneration and hyperplasia of endothelial cells may ensue after radiation, together with the formation of microthrombi, and these may cause capillary obstruction (Rubin & Cassarett,

1968). These authors have, however, noted that the vascular effects are decreased by the use of small and multiple radiation doses in experimental tumours, but these received incomplete tumoricidal doses. The inflammatory reaction associated with radiation damage results in the leakage of colloids and the deposition of fibrin (Law & Thomlinson, 1978), which may impair oxygen diffusion. Increased proliferation of endothelial cells in tumours has been documented (Denekamp, 1982) and as these cells are well oxygenated they may be relatively radiosensitive. The effects of radiation on the fine vasculature have been observed soon after radiation (*e.g.* Faiardo & Stewart, 1971; Law & Thomlinson, 1978; Kawamura & Fujiwara, 1973) in experimental systems and are frequently observed by pathologists in patients treated with planned preoperative radiation and surgery using conventional fractionation. These effects of radiation on tumour fine vasculature may impair reoxygenation towards the end of a course of fractionated therapy which is spread over 6 weeks.

The mechanism of reoxygenation cannot be fully explained by cell death and other factors, such as impaired metabolism of oxygen by radiation-sterilized, but still viable cells may play a role (Kallman, 1972). In the absence of this information the possibility that reoxygenation is a limited process in certain types of tumours must be considered. In the tumour model, reoxygenation is assumed to fail during therapy and a high proportion of hypoxic cells will subsequently rapidly develop with continued radiation.

MISO will be of great value in sensitizing these remaining hypoxic cells, as their sterilization will be critical to the achievement of tumour cure.

*MISO and radiation dosages.*—The sensitizer enhancement ratio of MISO is related to the concentration of the agent (Asquith *et al.*, 1974). The total dose of MISO which can be administered during a course of radiotherapy is, however, limited by the drug's neurotoxicity (Dische *et al.*, 1979). The major advantage of fractionation in terms of the reduction in the number of hypoxic cells, namely reoxygenation, is no longer applicable towards the end of therapy in the tumour model. The largest cell kill for hypoxic cells will thus be obtained by using MISO in high doses and with a few relatively large radiation fractions. In order to maintain a constant relative biological effectiveness (RBE) for the radiation fractionation schedule, which includes doses of radiation as compared to the fractionation schedule which it is replacing, the size and the number of large radiation fractions will require adjustment.

FIGURE.—Survival curve of chronic hypoxic cells in a tumour model with reoxygenation occuring up to 40 Gy and not thereafter. —— Hypoxic cells undergoing reoxygenation; — . — Remaining hypoxic cells +MISO (SER 2); - - - - Remaining hypoxic cells alone.

Large fractions of radiation will, however, adversely affect radiobiological repair of normal tissue cells and measures to prevent normal tissue damage will be indicated (see below).

The survival curves for the hypoxic cells in the tumour model are illustrated in Fig. 1. The Do for the hypoxic cells undergoing reoxygenation mirrors the Do of the oxygenated tumour cells, assuming that the optimal radiation fractionation schedule is being used? It will be negligibly affected by MISO administration. The Do for the remaining hypoxic cells which do not undergo reoxygenation will be approximately 3× greater. An arbitrary (sensitization enhancement ratio) (SER) of 2 has been used to determine the survival curve of the hypoxic cells irradiation in the presence of MISO. The large fractions of radiation used with MISO have been normalized to the fractionation schedule initially used for comparative purposes.

*Clinical applications*.—This tumour model may reflect the behaviour of certain tumours in patients, although it is obviously simplified and any transition from active reoxygenation to its termination will be more gradual.

A radiation fractionation schedule for use clinically together with MISO has been devised on the premises outlined above. It consists of an initial course of 2 Gy in daily fractions to a dose of 40 Gy over 4 weeks, which probably is an efficient fractionation schedule in the presence of reoxygenation in view of its widespread empirical adaptation. This is followed by 6 Gy given twice, one week apart together with high doses of MISO to a dose of 12 Gy. The interval of one week between the 2 large fractions is used in order to prevent increased normal tissue damage which may result from the 2 large fractions and is based on the reasonable expectation that the slowly dividing hypoxic tumour cells will repopulate to a lesser extent than the normal tissue cells. In addition coned down volumes can be used for the large fractions. This approach has been clinically since 1979 in pilot studies in the treatment of bladder carcinoma. In an initial pilot study MISO was used orally only, after which it was used in a second pilot study both orally and intravesically in order to boost further the tumour concentration of MISO. The dose of MISO used in the second pilot study was 3·0 $g/m^2$ orally 4 h before radiation.

Improvement in the complete response rate at cystoscopy in the treatment of T2 Grade III and T3 bladder carcinoma has been obtained in 22 patients treated with radiation plus oral and intravesical MISO compared with 63 patients treated by radical irradiation between 1972 and 1977 who served as historical controls (73% *vs* 43%—significant at 5% level of confidence—$\chi^2$ test). Improvement has also been maintained at a mean follow up of 13·3 months and there has been no MISO neurotoxicity in this pilot study. There have been no late radiation complications in either of the 2 pilot studies, 14 patients having been followed up for between 12 and 24 months (Abratt *et al.*, submitted for publication).

Alternative and rational approaches based on different premises to the clinical use of MISO have been suggested by others (Denekamp *et al.*, 1978, 1980). Therapeutic protocols which effectively use MISO are likely to be dependent on the tumour's pattern of reoxygenation. This has been found to vary in different tumour systems in experimental animals (Kallman, 1972). In the absence of this information in patients, clinical work is proceeding empirically to a large extent. Information on reoxygenation in human tumours will be of great value not only in the design of efficient radiation fractionation schedules but also the planning of therapeutic protocols which include radiosensitizers, high linear energy transfer radiation and hyperthermia.

I wish to thank Professor R. Sealy and Drs E. Herring, G. Blekkenhorst and W. Levin for stimulating discussions, and Roche Laboratories for their support.

### REFERENCES

Asquith, J. C., Watts, M. E., Patel, K. B., Smither, C. E. & Adams, G. E. (1974) Electron affinic sensitization V. Radiosensitization of hypoxic bacterial and mammalian cells *in vitro* by some nitroimidazoles and nitropyrazoles. *Radiat. Res.*, **60**, 108.

Denekamp, J., Fowler, J. F. & McNally, N. J. (1978) Hypoxic cell sensitizers: early or late in fractionated therapy? *Br. J. Cancer*, **37**, 858.

Denekamp, J., McNally, N. J., Fowler, J. F. & Joiner, M. C. (1980) Misonidazole in fractionated radiotherapy: are many small fractions best? *Br. J. Radiol.*, **53**, 981.

Denekamp, J. (1982) Endothelial cell proliferation as a novel approach to targeting tumour therapy. *Br. J. Canecr*, **45**, 136.

DISCHE, S., SAUNDERS, M. I., FLOCKHART, I. R., LEE, M. E. & ANDERSON, P. (1979) Misonidazole. A drug for trial in radiotherapy and oncology. *Int. J. Radiat. Oncol. Biol. Phys.*, **5**, 851.

FAIARDO, L. F. & STEWART, J. R. (1971) Capillary injury preceding radiation-induced myocardial fibrosis. *Radiology*, **101**, 429.

FOWLER, J. F., SHELDON, P. W. & DENEKAMP, J. (1976) Optimum fractionation of the C3H mouse mammary carcinoma using X-rays, the hypoxic cell radiosensitizer RO-07-0582, or fast neutrons. *Int. J. Radiat. Oncol. Biol. Phys.*, **1**, 579.

KALLMAN, R. F. (1972) The phenomenon of reoxygenation and its implications for fractionated radiotherapy. *Radiotherapy*, **105**, 135.

KAWAMURA, R. & FUJIWARA, K. (1973) Effects of irradiation on the fine vasculature of normal and malignant tissues. In *Fraction Size in Radiobiology and Radiotherapy*, (Ed. Sugahara *et al.*). Tokyo: Agaku Shoin Ltd. p. 27.

LAW, M. P. & THOMLINSON, R. H. (1978) Vascular permeability in the ears of rats after X-irradiation. *Br. J. Radiol.* **51**, 895.

RUBIN, P. & CASSARETT, G. W. (1968) *Clinical Radiation Pathology*. Philadelphia: W. B. Saunders.

SHELDON, P. W. & FOWLER, J. F. (1978). Radiosensitization by misonidazole of fractionated X-rays in a murine tumour. *Br. J. Cancer.*, **37** (Suppl. 3), 242.

TANNOCK, I. F. (1970) Population kinetics of carcinoma cells, capillary endothelial cells and fibroblasts in a transplanted mouse mammary tumour. *Cancer Res.*, **30**, 2470.

TANNOCK, I. & HOWES, A. (1973) The response of viable tumour cords to a single dose of radiation. *Radiat. Res.*, **55**, 477.

VAN PUTTER, L. M. & KALLMAN, R. F. (1968) Oxygenation status of a transplantable tumour during fractionated radiotherapy. *J. Natl Cancer Inst.*, **40**, 441.